# Supplementary material for: A Quality Improvement Project to Support Post-Intensive Care Unit Patients with COVID-19: Structured Telephone Support
Source: Int J Environ Res Public Health. 2022 Aug 6;19(15):9689. doi: 10.3390/ijerph19159689 (PMC9368104; doi:10.3390/ijerph19159689)
Supplement: Supplementary file 1 [file ijerph-19-09689-s001.zip › ijerph-1811403-supplementary.pdf]

Supplementary material

Table S1. Qualitative data quotes and categories ordered by PICS-framework.

Study number	Quote for symptoms	Additional symptoms Ordered by PICS	Additional symptoms other than PICS	Quote for interventions
1	*Since 1 week tongue can move less well to the left, swallowing is more difficult, talking is also less easy*<br><br>*Lost weight (15kg), now gained again*	1.2	Weight loss	*Advised to contact GP tongue, swallowing and speech*<br><br>*Receives physical therapy*
2	None	None	None	*Physiotherapy is going well, comes 1x pw to the house*<br><br>*Dietician is also involved. Have a phone consultation*<br><br>*No further action required for now*
3	*Patient cannot carry out his work as a lawyer because of fatigue and memory loss.*	2.1<br>2.2	Difficulty accepting the physical consequences after IC and COVID.	*Has completed a course within rehabilitation centre but this did not result in improvement. Now on the waiting list for another one. Follow-up telephone consultation: we keep in touch*
4	Patient still can't open jars<br><br>Patient still sore ribs after a fall in the hospital<br><br>Patient is still very emotional during conversations<br><br>Caregiver is more tired lately<br><br>Sleeping is worsening since the last days	1.2<br><br>3.1<br><br>4.1<br><br>4.1	Problems with ribs after falling in hospital	*GP has been a long time and this afternoon physiotherapy comes for intake*<br><br>*If this problems persists, she will contact the family doctor (informal care)*<br><br>*In the COVID care pathway the patient receive further follow-up*
5	Patient still can't open jars<br><br>Patient still sore ribs after a fall in the hospital<br><br>Patient is still very emotional during conversations	1.2<br><br>3.1<br><br>4.1	Problems with ribs after falling in hospital	*GP has been a long time and this afternoon physiotherapy comes for intake*<br><br>*If this problems persists, she will contact the family doctor (informal care)*

	Caregiver is more tired lately<br><br>Sleeping is worsening since the last days	4.1		*In the COVID care pathway the patient receive further follow-up*
6	None	None	None	*Started with physical therapy this week*<br><br>*Rehabilitation will call after 3 weeks for cognitional problems*<br><br>*No indication for further interventions at this moment. Patient will contact us if necessary and receives followed up in the regular aftercare program.*
7	*In his own words, he sometimes gasps for breath*<br><br>Patient thinks its stress-related. He lost 18 kg in total. Now 2.5 kg gained weight again<br>Started physical therapy since this week	3.1	Weight loss	*Patient still gets physical therapy 2x a week.*<br><br>*He has been getting speech therapy for 1.5 months.*<br><br>.
8	At night, nocturia and swollen feet<br><br>Can hardly make a fist on the right, no strength	1.3	Nocturia and swollen feet	*Three times a day physio exercises via video calling with a physical therapist*<br><br>*Possibly expand physiotherapy face to face, instead of video calling*
9	Recovery is developing very slowly, according to patient nausea + vomiting were limited factors.<br><br>Wound tailbone almost closed<br><br>Sleeping is a problem, waking up when going to the toilet, then unable to get back to sleep. As a result, patient is tired during daytime	1.3	Nausea and vomiting<br><br>Wound on bump	*Occupational therapy for hand coordination and strength*<br><br>*If no indication for inpatient rehabilitation, start physical therapy at home*<br><br>*Advice given on sleep hygiene*<br><br>*Regularly via aftercare clinic IC after 3 months*
11	Sleeping is usually good, sometimes awake in between, then a bit more tired during the day	1.3	None	*Yesterday started with physiotherapy*

				At this moment no need for telephone consultation, patient will receive follow-up care via ICU aftercare clinic
13	Sleeping is difficult due to pain in shoulder<br><br>Feels oppressive on exertion, but is calmed by the measured saturation, which is normal at that time.<br><br>Sets high goals for herself, sometimes finds it difficult that this is not always attainable. Keeps looking for balance.	3.1	Sleeping is difficult due to shoulder pain<br><br>Sets high goals for himself. Finds it difficult that this is not always achievable. Searching for balance.	*In process of rehabilitation at home with physio*<br><br>*Family receives psychological support in the ' first line'*<br><br>*Discuss sleeping problems with GP for a possible medication treatment*
14	Patient still has 1 major complaint that restrains him in daily function. He still has a bladder catheter in situ because of bladder retention.<br><br>Pain in right lung<br><br>He does find it difficult how to deal with visitors. Afraid of a recurrence of Covid19.<br><br>Also worried about the bladder catheter.<br>Now also uncertain about visitors.	1.1<br><br>3.1	The patient has 1 major complaint that hinders him the most. He still has a bladder catheter in situ due to bladder retention.<br><br>Patient expresses difficulties to deal with visitors because of fearing a recurrence of Covid19.	***For now I arranged a phone appointment next Monday, May 25 with urologist***<br><br>***2 times a week physiotherapy.***<br>***No additional support needed.***<br><br>***In July appointment with pulmonologist for CT scan.***<br><br>***If patient continues to have cognitive symptoms in the longer term report to GP.***<br><br>***Advice given to adhere to the RIVM guidelines.***<br><br>***Advice given to look at www.ICconnect. Indicated that they will receive a letter from the aftercare clinic. Email-address also given.***
15	Patient has still al lot of thougts at night, only sleeps, sleeps an average of 2 to 3 hours a night<br><br>Patient becomes emotional several times<br><br>Nightmares, thoughts of the delirium he experienced. Lying tied up, wanting to	3.1<br><br>3.2	Patient gets emotional several times during meeting.	***Discussed consideration of more muscle strength building with physiotherapy. Referral for physio sent to home address so it can still be used at home if necessary.***<br><br>***Additional support is recommended.***

	take out the breathing tube. He understands that this was necessary but now has unpleasant thoughts about it which continue to haunt him			***Psychological help is needed.***<br><br>***I will call the GP of Mr. and Mrs.***<br><br>***Outpatient dietician still in consultation.***<br><br>***Monday 25-5 another TFU scheduled to see what the situation is then.***
16	Voice still a bit hoarse<br><br>Shaky hands still present	1.2	Voice is still a bit hoarse	***Physiotherapy 2 times a week.***<br><br>***This reduced (trembling hands) but if this persists discuss with physio/homeopathic.***
17	None	None	None	***Physiotherapy two times a week***<br><br>***Psychologist is involved, Is on the waiting list for the psychologist. Has the time wait for this***
18	Symptoms of dizziness<br><br>Patient is sometimes impatient. And that sometimes causes unrest fort he rest of the family.<br><br>Burden of the caregiver. Complains about the impatience of the patient and relational issues because of this.<br><br>Fatigue, would like support in this.	1.3	He is sometimes impatient. This sometimes causes for unrest in the house.<br><br>Overburdening informal caregiver. Exchange of words about the impatience of the patient.<br><br>Tiredness informal caregiver, would like support	***Physiotherapist is visiting patient at home***<br><br>***I ask social work for advice, how to handle this in the first line.***<br><br>***I have also indicated that time is also an important factor. That it is also a natural progression that dependency can bring tension into a relationship. But they are clearly asking for help so I am going to find out and we will link back to each other on Monday via a video consultation.***<br><br>***Translated with www.DeepL.com/Translator (free version)***
19	Patient still experiences a hoarse voice	None	Voice is still hoarse	***"Dad app" read was helpful to process period in Germany. No need for***

	Caregiver is sometimes tired		Informal caregiver is tired sometimes	***additional psychological support.***<br><br>***Ergotherapist is visiting soon.as also the Physiotherapist in the ' first line' now being taken up from 25-6, Urology 25-6 day admission at the hospital for bladder retentions and dietician calls in 2 weeks.***<br><br>***Follow-up telephone consultation: no indication for now, regular follow-up is initiated.***
20	The phone conversation is intense because of the low energy and emotional burden.	2.1	Feeling sad during rehabilitation process	***Gets physiotherapy 2 times a week.***
	Difficulty finding words	2.2	Is curious about recovery, sometimes feels insecure about it	***Home care once a week in the morning***<br><br>***Mrs. called her GP herself for psychological help at home instead of going to the hospital.***
	Often sad about the rehabilitation process			
	Is curious about recovery, sometimes feels insecure about it			***In 2 weeks a follow-up consult via video call is initiated, after consult to evaluate if there is progress in recovery.***
			Follow up<br>Difficult moments when recovery is taking so much time	***Follow up-consult***<br>***Physio and psychology is involved.***<br><br>***Been to the lung specialist for a check-up and will inform for a thoracic CT***
	Follow up consultation<br>Difficult moments that recovery is so slow			***I give the advice to write down difficult moments so that she can discuss these at the time of consultation.***<br><br>***The patient was videotaped during the hospitalization. She has not heard anything about informed consent***

				about this. I will contact communication staff about this matter.
21	Patient reports to sleep poorly, has to get out of bed often due to nocturia.<br><br>Since Tuesday, 2 episodes of cold shivers with hypothermia; 34.0 Celsius.	None	Sleep difficulties, has to get out of the bed often due to nocturia.<br><br>Since Tuesday two times cold shiver with under temperature of 34C	***Physiotherapist specializing in COVID-19 is involved and will start in 6 weeks.***<br><br>***If a new episode of cold shiver and hypothermia is present, I advised to contact the GP.***<br><br>***No need for follow-up consultation***
22	Patient is suffering from a sleeping disorder<br><br>Feels abandoned because of his wife's leaving and wonders what to do next	3.2	Feeling abandoned because of his wife's departure and is curious about how things should continue in future	***Crisis service is involved due to nights not sleeping. Family physician is also involved. We will call***<br><br>***Physiotherapist is involved.***<br><br>***At this moment, we cannot deploy additional actions at this time.***
23	The patient is particularly preoccupied with the fact that her partner has infected her with the corona virus<br><br>She notices that her concentration speed is difficult and that she is sometimes unsure of her memory<br><br>Afraid of getting COVID-19 again and difficulty in relating to her partner in view of the infection<br><br>Often has thoughts about the ICU admission and the fear that was associated with it.	2.2<br>2.5<br><br>3.2	The patient is mainly concerned with the fact that her partner infected her with the corona virus<br><br>Afraid to get corona again and she is having difficulties with the relationship to her partner since he gave her the infection	***Klinische revalidatie zorgt voor juiste programma en structuur***<br><br>***Nutritional status is optimal with the supplementary nutrition's: nutridrinks***<br><br>***The patient had conversations about coping with partner.***<br><br>***Social work is involved with these problems.***<br><br>***Thoughts of IC can decreases by time. At this time, no further action is needed.***<br><br>***Disciplines involved: dietician, social work and physiotherapist***<br><br>***Advice for this patient is to continue as before and visit the IC aftercare clinic***

24	Patient continues to have pain in the hip (right)<br><br>Fingers feel stiff on both hands. Not numb<br><br>Oedema in both legs are still present<br><br>Insertion trachestoma still patchy, still leaking some serous fluid.<br><br>Still afraid of recurrence.<br><br>Anxious son, doing better, but very fearful of a recurrence of COVID-19.	4.1	Patient still has pain in his right hip<br><br>Fingers feel stiff on both hands. Not insensible.<br><br>Oedema is both legs still present<br><br>Insertion of the tracheastoma is still having a plaster, still leaking some serous fluid<br><br>Anxious son, doing better, very afraid of his fathers' health and the possibility of him getting a relapse of COVID.	***Orthopaedics is involved***<br><br>***Physiotherapist is involved for three times a week for 30 minutes***<br><br>***GP is involved. Has given stockings for oedema***<br><br>***No inflammatory signs. No fever.***<br><br>***Dietician does a phone consultation***<br><br>***GP is aware of this***<br><br>***Information given about aftercare clinic, visit would be nice for patient in a while.***<br><br>***www.icconnect.nl passed on as information source for e.g. IC cafe***
25	Sometimes has pain during breathing<br><br>Dreams, but still present but less intensive.<br><br>Wondering if a full recovery is feasible.	3.2	Sometimes pain during breathing<br><br>Curious if she will recover fully	*Physiotherapist is involved*<br>*26-5 appointment with GP to discuss complaints*<br><br>*Contacting the GP about the complaints is recommended*
26	A bit slower thinking suited to the situation, is the patient' opinion.<br><br>Does worry about the progress of CLL	2.5	Worried about development CLL	***Picking up my daily activities and go to physiotherapy.***<br><br>***Monday I had a blood test and now Wednesday 6-5 I have the results by phone***

	Stresses from ICU admission, experiences problems communicating with family (informal care)	4.2	Patient experiences stress from memories of the ICU admission, problems in communication with his family members (informal caregivers)	***and a conversation with a lung specialist.***<br><br>***Spouse needs conversation, victim support referred to IC connect but this did not help. Interview with spouse scheduled via video consultation to provide a listening ear for tension and IC experience of spouse and to draw up plan for this burden.***<br><br>***Follow up consultation:***<br><br>***Reconsider additional help. But no need for additional interventions. If more help is needed conact with GP or send email to our mailadress: xxxxx@xxxxxxx***<br><br>***Outpatient appointment with pulmonary medicine is arranged through the outpatient clinic, not received a invitation yet.***
27	For a while, it was disappointing that he was very dependent of others.<br><br>Tingling right hand and right foot<br><br>Partner feels tired but do can find balance	1.2	It was disappointing that he was very dependent<br><br>Wife feels tired, but can find balance	***Patient had referral physio, but did not deploy it, thought he would be contacted, but this turned out not to be the case. Patient contacts GP and goes to request physio***<br><br>***- Follow-up consult -***<br>***Brief telephone contact to check whether physio was successful.***<br><br>***Patient indicates that this is the way to proceed with recovery, agree on this.***
28	Fatigue, partly because the patient sleeps differently. Still has regular dreams, but these are decreasing in intensity	3.2		***There is progress with the help of physiotherapy.***<br><br>***Upon discharge, I was referred for 1st line occupational therapy.***

	Still reduced feeling/pain dig IV/V and ulnar side left forearm DD with ulnaropathy<br><br>Patient indicates that daughter has problems with coping, does not speak about anything that is happened.	1.2<br><br>4.4		***However, no action has been taken on this yet due to transport problems.***<br><br>***Advised to still contact occupational therapy to see if they have any additional advice with regard to right hand***<br><br>***No telephone consultation, but certainly continue in aftercare program. Attention for psychological burden, also for daughter.***<br><br>***A Polish-speaking psychologist should contact after returning from maternity leave if dreams/anxiety are limiting factor in recovery.***
29	Respiratory problems still present, especially during exercise.<br><br>Showering and climbing stairs still requires a lot of energy<br><br>Sometimes a stabbing pain in the thorax<br><br>In the beginning nightmares and fears, both become less present.	1.3<br><br>1.3<br><br>3.2	Sometimes stabbing chest pain	*Physio starts 10-12-20 If stabbing pain persists contact your doctor*<br>*Respiratory problems still quick on exertion*
30	Reported pain in shoulder and throat<br><br>Sleeps poorly, sleeps 2 hours a nights.		Pain in shoulder and throat<br><br>Sleep difficulties, sleepts 2 hours a night	***Physiotherapy and showering 3 times a week. Daily assistance in the morning***<br><br>***Sleeping badly and the pain is discussed by the patient with the GP***
31	Still low oxygenation at home, especially in the night still saturations up to 89 - 90%. Needs addition 1L oxygen.<br><br>Taste is not yet recovered fully.		Still oxygen at home, especially at night still saturations up to 89 – 90%, on and off 1 liter of O2.<br>Taste is not right yet	***Medio March an appointment is made with a lung specialist, as also a CT thorax is scheduled.***<br><br>***if other symptoms persistent or new symptoms are developing, I advice to return to GP for analysis. If***

	Sleeps badly, can't sleep or wakes up to go tot he toilet.		Sleeps badly, can't sleep or wakes up to go to the toilet.	***recovery is stagnant, consider involving the rehabilitation practitioner again.***
32	Depressed feelings, psychologist is involved.	3.3	None	***Physiotherapy twice a week***<br><br>***Monday the general practitioner comes to see the patient; the patient will discuss a follow-up of the blood pressure and glucose regulation.***<br><br>***Son contacts a psychologist patient where the patient was treated before the ICU admission***
33	Swallowing is still difficult, but does not choke<br><br>Indicates that he is sometimes more/quicker irritated	1.2<br><br>3.2		***Home care supports daily support with taking a shower.***<br><br>***The following practioners are involved: Physiotherapy, home care for ADL, speech therapy and dietetics***
34	Less agitated, more relaxed than before, no longer so worried		Less agitated, more relaxed than before, no longer worried about things	***Yesterday I started rehabilitation. Tomorrow, there is an interview with rehabilitation doctor to map out physical complaints.***
35	Short-term memory is somewhat reduced, this improves<br>Less agitated, more relaxed than before, no longer so worried	2.2	None	***Physiotherapist is involved***<br><br>***Dietician is involved***<br><br>***The family doctor is also involved***<br><br>***No follow-up consultation planned. I have shared the email addresses of our service : xxxx@xxxx***
36	Patient suffers from swollen feet, especially during the day<br><br>Husband sleeps only 1 hour per night, because of dreams of the ICU.	4.2	Patient suffers from swollen feet, especially during the day	***Receives home care twice a week for help with showers***<br><br>***Occupational therapy comes on Monday for possible adjustments in the bathroom (brace etc)***<br><br>***Husband has already seen his GP and been given a***

				sleeping tablet. Not open to professional help.<br><br>***Tomorrow there is contact with GP, the patient will discuss swollen feet***
37	Avoidance of memories, however. In the beginning often awake, no obvious nightmares. Slowly becomes less.<br>Still suffers from trembling hands on exertion. Was present before ICU admission.	3.2<br><br><br><br>1.2		***Physiotherapy involved for two times a wee.***<br><br>***Rehabilitation aftercare is initiated.***
38	X	X	X	***X***
	Mr. has sufficient support in the environment such as family, physio and dietician for further recovery	1.1<br><br><br><br><br><br>3.1	Notices that he wants more than he can	***There is support in the environment such as family, physiotherapist and dietician for further recovery***<br><br>***No follow-up consultation.***
40	Higher blood pressure	None	Sleeping is variable	***General practitioner is involved for blood pressure***<br><br>***involved: physiotherapy***<br><br>***No more check-up appointments in the hospital are made.***
41	mainly burden: loss of strength	1.3	None	***Physiotherapy not yet possible due to positive test of COVID-19 of son Will be continued.***
42	Still needs oxygen<br><br>Sleeping problems: falls asleep with difficulty. Sleeps 3 - 4 hours/day.<br><br>Diabetic mellitus as new diagnose	1.1	Sleep problems; difficult to fall asleep. Sleeps 3 – 4 hours a day.<br><br>New diagnosed with diabetic	***Physiotherapy still to be started***<br><br>***General practitioner, lung specialist are involved***
43	Swollen feet since this weekend<br><br>Sleeping is badly, due to dyspnoea		Since last weekend swollen feet<br><br>Difficulties with sleeping due to dyspnea	*I will follow up on the doctor's discharge letter and follow-up appointments. Advised to contact the general practioner and possibly the lung specialist in the event of further deterioration of the respiratory system*
44	Especially very anxious to get COVID-19 again. Has no contact with people. Has	3.1	None	***Physiotherapy twice a week***<br><br>***Psychologist is involved***

	little confidence in people she does not know. Gets angry with husband if he lets people in. Son and husband come home from school or work and have to take a shower and put on clean clothes.			***social worker is involved to support the family***<br><br>***advice on protein-rich diet. Dietician had completed the consultation***<br><br>***Email address shared if there are any more questions left.***
45	Pain around thorax lessening<br><br>Still a lot of loss of muscle strength, especially in the arms	1.3	Pain around thorax is decreasing	*Advice to patient*<br>*1. The patient should call in the physiotherapist at an early stage.*<br>*2. Contact GP if recovery is not progressing.*<br>*3. Explanation given: www.icconnect.nl for more information.*<br><br>*Planned: Patient has another check-up at cardiology on 27-11*
46	Follow-up<br>Patient explaines he sleeps poorly and remembers everything that happened in the ICU very well. Has experienced this very consciously. Was therefore sometimes anxious at the thought of perhaps being put to sleep and being put on a ventilator.<br><br>Due to sleeping badly, the patient experiences memory problems and is easily disturbed.<br><br>Patient feels gloomy when sleeping badly<br><br>Negative thoughts about being admitted to the ICU at night. Thoughts of perhaps not being able to see her grandson and possibly having to be put on a ventilator.	3.1<br><br>3.1<br>2.2<br>2.5	None	***Had contacted the IC twice because of these complaints.***<br><br>***The company doctor had also advised her to seek psychological help.***<br><br>***Physiotherapist called in herself.***<br><br>***Lung specialist Machiels was sent a basket with the request to request a psychology consultation.***<br><br>***Follow-up telephone consultation: 14 Dec follow-up consultation to discuss further actions.***<br><br>***Follow-up***<br>***Patient has requested physiotherapy in order to work on her condition.***<br><br>***Appointment at medical psychology is made on 5***

		January and shared with the patient.
	3.3	
		Patient can continue his way and may call in case of problems
	3.1	
